# Chondroprotective Factors in Osteoarthritis: a Joint Affair

**DOI:** 10.1007/s11926-019-0840-y

**Published:** 2019-06-21

**Authors:** Jolet Y. Mimpen, Sarah J. B. Snelling

**Affiliations:** The Botnar Research Centre, Nuffield Department of Orthopaedics Rheumatology and Musculoskeletal Science, University of Oxford, Oxford, OX3 7LD UK

**Keywords:** Chondroprotection, Osteoarthritis, Stratification, Drug development

## Abstract

**Purpose of the Review:**

Osteoarthritis is widely regarded as a spectrum of conditions that affect all joint tissues, typified by a common entity: cartilage loss. Here, we review recent progress and challenges in chondroprotection and discuss new strategies to prevent cartilage loss in osteoarthritis.

**Recent Findings:**

Advances in clinical, molecular, and cellular characterization are enabling improved stratification of osteoarthritis subtypes. Integration of next-generation sequencing and “omics” approaches with clinically relevant readouts shows promise in delineating both subtypes of disease and meaningful trial end points. Novel delivery strategies are enabling joint-specific delivery.

**Summary:**

Chondroprotection requires a whole joint approach, stratification of patient groups, and use of patient-relevant end points. Drug development should continue to explore new targets, while using modern technologies and recent knowledge to re-visit unsuccessful therapeutics from the past. The overarching goal for chondroprotection is to provide the right treatment(s) for the right patient at the right time.

## Introduction

Osteoarthritis (OA) is a highly prevalent and extremely disabling condition, affecting 30.8 million people in the USA alone [1]. In 1741, J.B. Morgagni first described articular cartilage loss in OA [2]. To this day, radiographic measurement of cartilage loss remains the key diagnostic tool for OA [3]. Consequentially, chondroprotection has been a major focus of disease modifying treatment in OA. Chondroprotective drugs (disease modifying OA drugs, DMOADs) have generally targeted structural changes of disease by aiming to inhibit cartilage-degrading factors or promote cartilage production, with or without effects on symptoms. Of the $81 billion annual healthcare expenditure on OA in the USA, 15% is attributed to prescription drugs [4, 5]. However, an effective means to prevent OA-related damage has proven elusive. Therefore, much of this expenditure centers on drugs that target pain rather than structural changes. Given current limitations in DMOADs, OA progresses and surgical joint replacement is the only viable option. However, joint replacement is by no means a panacea with a mortality rate of 1% and at least 10% of total hip or knee replacements reporting no improvement or worse symptoms at 1 year after surgery [6, 7].

In this review, we consider recent progress and challenges in chondroprotection and how chondroprotection should encompass both direct (within cartilage) and indirect (articular or systemic) strategies. We also discuss how a step change in successful chondroprotection requires reframed thinking in disease stratification, target identification, and drug development, including drug delivery strategies.

## Osteoarthritis—Chondroprotection Is Not Chondrocentric

**Fig. 1:**
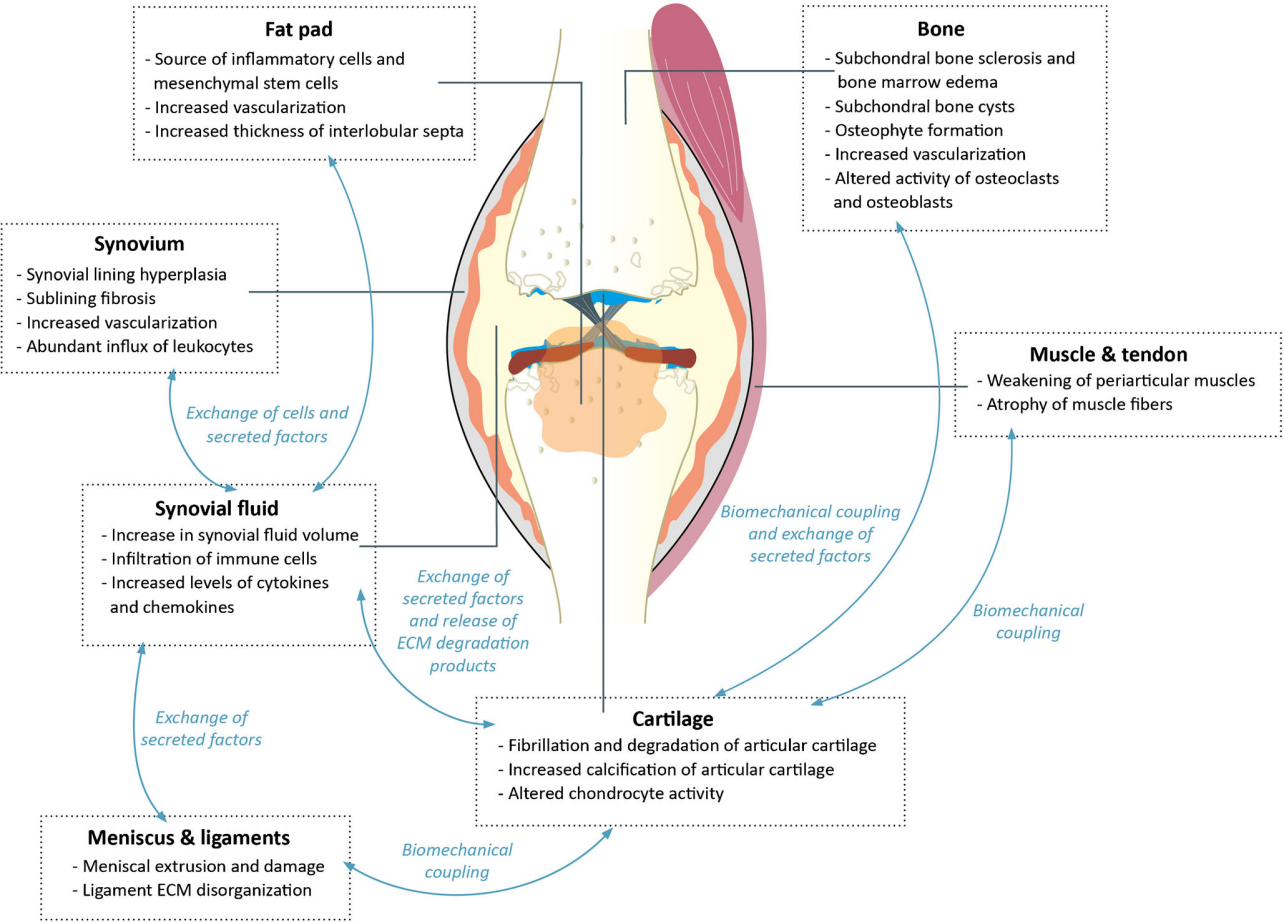
A representation of an osteoarthritic knee, including the main contributing tissues and their interactions. The exact balance of tissue involvement and interaction is dependent on both joint site and the subtype of OA. Articular cartilage loss typifies OA but the exact balance of tissue involvement and interaction is dependent on joint site, stage and subtype of disease

Osteoarthritis (Fig. 1) occurs at synovial joints and was long regarded as a structural disease of wear and tear. The key symptoms of OA are pain and joint instability, with radiographic joint space narrowing due to cartilage loss being a major clinical sign of disease. Alongside OA-related cartilage degradation, osteophytes form at joint margins, and bone remodeling occurs, leading to bone marrow lesions and bone sclerosis [8]. Synovitis (synovial inflammation) and meniscal damage are common and altered levels of inflammatory mediators are detected in OA synovial fluid. Furthermore, OA is now widely accepted as a whole joint disease [9], but the chronicity of tissue involvement in OA remains ambiguous, and cartilage may not be the initial site from which OA propagates. The cross-talk between cartilage and other tissues suggests cartilage loss can occur secondary to other OA-related changes to the joint [10]. We must also remember that synovial joints are, in fact, comprised of diverse tissues that experience different loads; have distinct functional requirements; and possess differing proportions of tissue types. The interactions of these factors likely explain the predilection of OA for certain anatomical sites (commonly knee, hip, spine, hands, and feet).

Chondroprotection offers a preventive strategy that might decelerate structural disease progression and loss of function and, through the manipulation of inflammatory pathways, improve pain. However, the success of such strategies demands a holistic approach that judiciously targets OA pathobiology across all joint tissues.

## Current and Recent Targets in Chondroprotection

Over the last 5 years, the quest for successful DMOADs has remained substantial. Recent DMOAD trials (Table 1) encompass chondroprotective factors and drugs targeting inflammatory cytokines, signaling mediators, senescence, and pain. From these studies, it is clear that antibodies against inflammatory mediators like IL-1 and TNF-α have failed in recent OA clinical trials. However, an exploratory analysis of the recent CANTOS trial demonstrated that IL-1 antibodies did reduce the incidence of joint replacements in patients with a history of myocardial infarction [15, 16]. A recent review by Chevalier and Eymard hypothesizes that IL-1 inhibition might not be effective at end-stage OA and, instead, earlier-stage disease or patients with systemic inflammation-related comorbidities may be more amenable to treatment. They also propose that non-intravenous routes of administration should be considered to improve bioavailability [48].

**Table 1:** Recent disease modifying osteoarthritis drug trials, including category, drug class, drug name, and description of results. NCT numbers of clinical trials are included where relevant. TNF = tumor necrosis factor, WOMAC = Western Ontario & McMaster Universities Osteoarthritis index, IL = interleukin, MDM2 = mouse double minute 2 homolog, MMP = matrix metalloproteinase, ADAMTS = a disintegrin and metalloproteinase with thrombospondin motifs, MEPE = matrix extracellular phosphoglucoprotein, RANKL = receptor activator of nuclear factor kappa-B ligand, BMP = bone morphogenetic protein

Category	Drug class	Drug	Description
Anti-inflammatory	TNF-α inhibitors	Adalimumab	Randomized open-label study showed it was effective and well tolerated [11]. Randomized double-blind placebo-controlled trial showed no difference in pain or synovitis in hand OA [12].
		Infliximab	One phase IV trial showed improved WOMAC scores [13]. No new studies or trials published since then.
		DLX105 single-chain scFV antibody fragment against TNF-α	Phase I clinical trial completed in 2010 (NCT00819572), results not published.
	IL-6 receptor inhibitor	Tocilizumab	Early studies show improved pain and morning stiffness [14]. Phase 3 clinical trial completed in February 2019, awaiting results (NCT02477059).
	IL-1 inhibitor	Gevokizumab (anti-IL-1β)	Results from phase II studies in erosive hand OA did not show a greater improvement than placebo (NCT02293564, NCT01882491, and NCT01683396).
		Canakinumab (anti-IL-1β)	CANTOS study showed fewer reports of OA with canakinumab than placebo [15]. Subanalysis showed a reduced number of total knee and hip replacements in the treatment groups than in the placebo group [16]. No new trials registered to date.
		Lutikizumab (anti-IL-1α/β)	Phase II study showed limited improvement of pain and lack of synovitis improvement [17].
	IL-1Ra inhibitor	Sc-rAAV2.5IL-1Ra	Trial to started recruiting in March 2019 (NCT02790723).
	p38 inhibitor	ARRY-371797	Phase II trial completed in 2012, but no results published (NCT01366014). No further trials conducted.
	Wnt inhibitor	SM04690	Pre-clinical studies showed anti-inflammatory and cartilage protecting effects [18–20]. Results from a phase II study showed significant symptomatic improvements and increase in joint space width [21]. Different further clinical trials are ongoing (NCT03727022 and NCT03706521).
	IκB kinase inhibitor	SAR113945	Phase I trial show promising results but larger patient sample is needed to show efficacy [22]. Phase II study completed but no results published to date (NCT01598415).
Senescence	p53/MDM2 inhibitor	UBX0101	Phase I trial in patients with knee OA due for completion in 2019 (NCT03513016).
Inhibition of cartilage-degrading factors	MMP-inhibitors	Doxycycline (non-specific inhibitor)	Randomized, placebo-controlled, double-blind phase III trial showed doxycycline slowing down joint space narrowing in the index knee [23]. However, a triple-blinded randomized controlled trial did not show any reduction in symptoms but did show an increase in adverse effects [24].
	ADAMTS-inhibitors	Anti-ADAMTS-5 nanobody	Currently awaiting results from phase I clinical trial (NCT03224702) after in vitro studies showed protection against cartilage breakdown [25]. Phase I clinical trial of multiple ascending doses of anti-ADAMTS-5 nanobody in knee OA, due for completion in May 2019 (NCT03583346).
	Anti-protease	Alpha-2-macroglobulin	Phase I trial to look at the reduction of pro-inflammatory synovial fluid biomarkers in OA due for completion in 2019 (NCT03656575).
	Cathepsin K inhibitor	MIV-711	MIV-711 was well tolerated in a phase I study in healthy subjects [26]. Phase IIa trial showed significant reductions in bone and cartilage disease progression in the femur [27]. No further studies registered to date.
Promotion of cartilage building factors	Fibroblast growth factor (FGF)	Sprifermin (rhFGF-18)	After positive results in pre-clinical trials [28, 29], the results from the first-in-human clinical trial were cautiously optimistic [30]. A phase II study to further investigate safety and effectiveness is due for completion in May 2019 (NCT01919164).
	Sulfated glucosaminoglycan/precursor of glycosylated proteins	Chondroitin sulfate and glucosamine	Many clinical trials have been conducted, most of which show mixed results for chondroitin sulfate, glucosamine, as well as the two in combination [31, 32]. However, a meta-analysis has shown that chondroitin could alleviate pain and improve function and that glucosamine improved stiffness [33].
	Hyaluronic acid	Intra-articular hyaluronic acid	Meta-analysis on the effect in hip OA did not show any difference to placebo [34]. However, a meta-analysis on knee OA showed a moderate but real benefit for these patients [35]. There are many active clinical trials that compare to hyaluronic acid with placebo, PRP, or other treatments, such as NCT03852914, NCT03801564, and NCT03690232.
	MEPE derivative	TPX-100	Phase II study showed it was safe, well tolerated, and associated with significant and clinically meaningful functional benefits [36]. No further studies registered to date.
Pain	Nerve growth factor (NGF)	Tanezumab	After a successful phase I trial [37] and an earlier successful proof-of-concept study [38], now many studies in progress awaiting results.
		Falranumab	Phase II double-blind placebo-controlled trial showed positive results on pain but risk of rapid OA progression [39]. Several phase III trials were finished in 2016, but no results have come out yet.
		Fasinumab	Randomized, double-blind, placebo-controlled trial showed improvement of pain and function, while generally being well tolerated [40]. Several phase III trials are currently recruiting patients (NCT02683239, NCT03304379, and NCT03161093), as well as a trial to self-administer fasinumab (NCT03491904).
	Trans-capsaicin	CNTX-4975	Phase II revealed that a single injection improved pain with walking, knee stiffness, and physical function in OA patients with knee pain [41]. Several phase III trials (NCT03661996, NCT03660943, and NCT03429049) are currently recruiting or completing their study.
	Neurotoxic proteins	Botulinum toxin A	Phase II trials show that intra-articular injection provided pain relief and improved functional abilities in knee OA patients [42, 43]. Multiple studies are currently investigating this effect further (NCT02832713 and NCT03187626).
Repurposed drugs	RANKL inhibitor (osteoporosis)	Denosumab	Denosumab reduced early migration in total knee replacement, which often causes the need for a revision [44]. Phase II trial is currently looking at denosumab in hand OA (NCT02771860).
	Calcium-reducing hormone	Calcitonin (osteoporosis drug)	Two phase III trials did not show any clinical benefits to patients with symptomatic knee OA [45].
		Anti-calcitonin gene-related peptide (migraine drug)	Phase II study was terminated as interim assessment showed lack of efficacy [46].
	BMPs	BMP-7	Phase I trial showed a symptom response and no dose limiting toxicity [47]. However, no further studies have been done.

Senescence is a compelling recent target in OA and other diseases of aging. Senescent cells are resistant to apoptosis and secrete inflammatory and catabolic mediators including IL-1, IL-6, and MMP-3. In OA mouse models, senescent cells accumulate in the synovium and cartilage; the small molecule UBX0101 clears senescent cells and ameliorates cartilage damage [49]. Clinical trials are now underway to test UBX0101 for amelioration of painful patellofemoral OA. In addition, repurposing of drugs, like those for osteoporosis, have received particular attention in the OA field. These drugs might serve as potential treatments in subtypes of OA, as osteoporosis and OA have commonalities in bone involvement [50]. Although repurposed Osteoporosis drugs show compelling results in pre-clinical testing in OA, clinical trials in late-stage OA, such as multiple phase III trials investigating calcitonin, have not yet demonstrated obvious benefits [45]. Galcanezumab, a monoclonal antibody targeting calcitonin gene-related peptide, was also unsuccessful in a phase II trial as it did not reduce signs or symptoms of knee OA [46].

Active research into surgical and cell-based strategies continues, but an exhaustive review of the current research landscape is not the purpose of this review. Rather, we seek to present a framework of principles that are applicable to future success of all chondroprotective approaches.

## Disease Stratification—Breaking Down a Spectrum of Diseases into Different Subtypes

Advances in clinical, molecular, and genetic characterization of OA suggest it is better regarded as a spectrum, or continuum, of conditions leading to end-stage disease with a common entity: loss of articular cartilage [51]. This is unsurprising given the varied clinical manifestations of OA. However, most current clinical and laboratory-based studies do not consider disease subtypes in their study design.

**Fig. 2:**
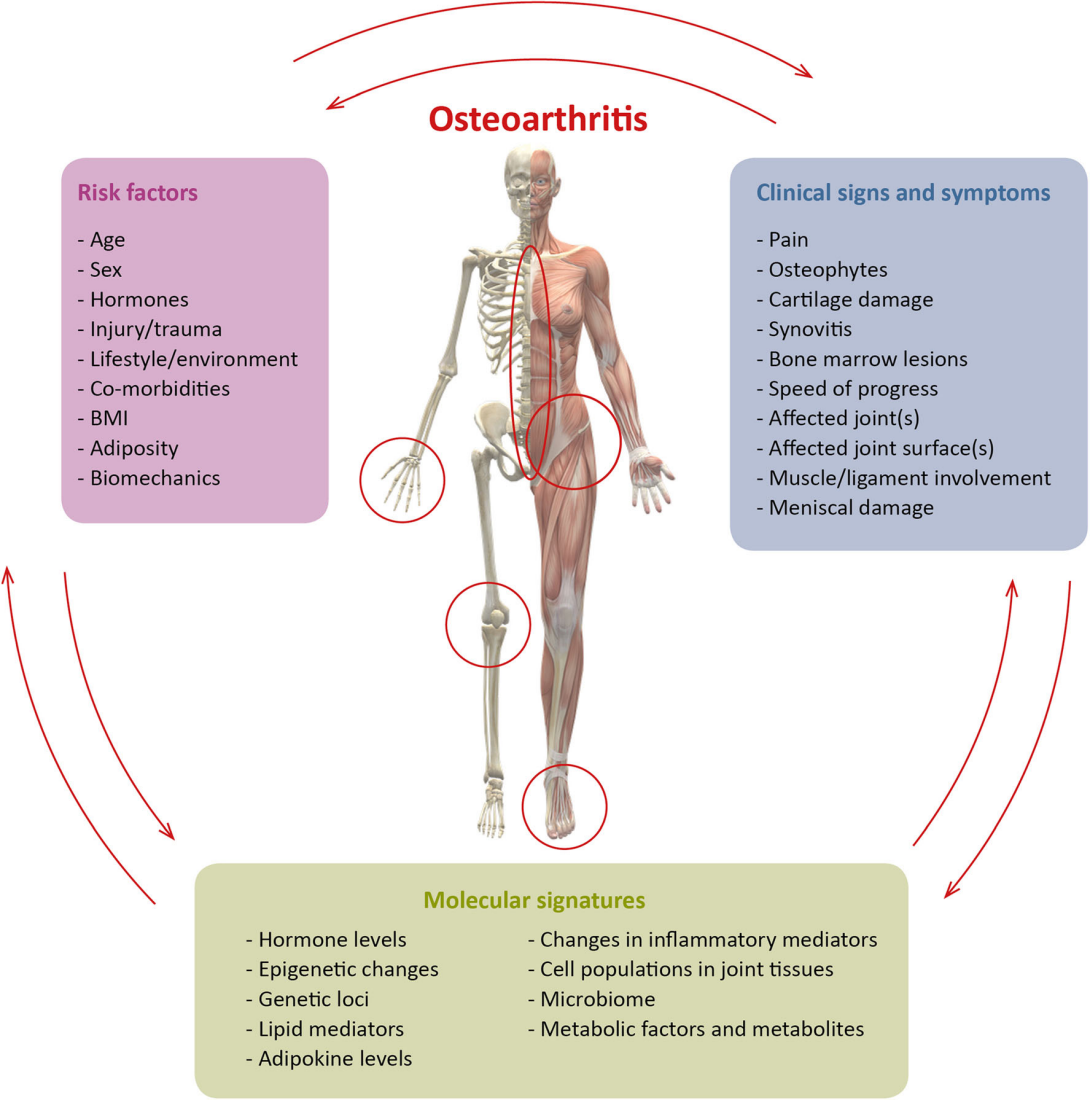
Overview of OA stratification categories and their interactions. Clinical signs and symptoms, risk factors, and molecular signatures interact and can define OA subtypes. The combinatorial effects of different stratification measures will define distinct subtypes and stages of OA. For example, risk factors such as trauma will drive changes in molecular and cellular signatures and altered clinical signs and symptoms. As OA progresses stratification categories will continue to feedback on each other, therefore individual stratification measures (or combinations thereof) may be specific to a particular stage of disease

Disease stratification is best demonstrated in oncology, where the ability to utilize molecular information to predict prognosis and optimal therapeutic strategy has been the first step towards personalized treatment [52]. To apply this to chondroprotection in OA, we must consider the key characteristics by which to stratify patients (Fig. 2).

### Clinical Phenotypes

A first step to classify OA can be through the affected joint(s). Clinically adopted imaging, pain, and function scores can also incorporate affected joint surface(s) and stage of disease. There are several studies where these scores reveal patterns in chondroprotective response. Individuals with severe cartilage loss have a reduced likelihood of improved pain following intra-articular steroid injection [53, 54]. Meanwhile, strontium ranelate inhibition of cartilage loss was more effective in patients with meniscal extrusion and bone marrow lesions than in those with meniscal extrusion alone [55]. Osteophytes and synovitis may also predict the rate of structural progression [8, 56], which varies between individuals, influencing both prognosis and chondroprotective strategy [57]. Potential predictors of structural progression include pain [58], inertia [57], bone marrow lesions [59], joint loading [60], baseline Kellgren–Lawrence (KL) grade, obesity, and alignment [61]. However, few studies have the power and quality to reliably deconvolute clinical predictors of cartilage loss or other clinical outcomes [62].

### Risk Factors

Risk factors for OA, including age, sex, and trauma, may hint at the underlying mechanism and determine the broadly applicable stratification steps. Age is associated with inflammaging and senescence and predicts improvement in pain following corticosteroid injection for knee OA [54]. Mechanisms underlying pain differ in males and females, and, consequentially, the mechanisms underlying cartilage damage may also be modulated by sex. Younger males are less likely to require joint replacement [63], and morphological differences between sexes might contribute to OA development and severity [64, 65]. Although few studies have investigated the effects of OA risk factors on pharmacologic driven chondroprotection, there is evidence that OA progression is reduced in post-menopausal women undergoing hormone replacement therapy [66, 67]. Site- and hormone-based interactions are also seen in the presentation of hand OA at the time of menopause, in contrast to other forms of OA that present post-menopause [68, 69]. Comorbidities, including obesity, diabetes, and metabolic syndrome, are additional risk factors for OA with clinically related readouts such as high serum cholesterol, which is associated with generalized OA [70]. Lifestyle, diet, and alteration of the microbiome may also affect OA onset and progression [71].

Joint loading and trauma significantly impact OA development. Lower step rate is associated with a greater risk of increased cartilage damage in knee OA patients [72]. Following traumatic ACL injury, initial cartilage damage is more common in the lateral and medial compartments of the knee than in the medial alone [73]. Sport-related knee injuries lead to a significantly greater likelihood of joint replacement [74], yet younger patients have differing underlying biology and immune status, higher physical demands, and greater need to avoid surgical joint replacement. These factors greatly impact the requirements of therapeutic chondroprotection while identifying a relatively defined group on which to test DMOADs [75].

The breadth of OA risk factors and clinical phenotypes demonstrates the wealth of potential disease subtypes. Risk factors alone, although identifiable prior to clinical presentation for OA, are not adequate to reliably predict OA occurrence or prognosis. A key advantage of stratifying OA patients based on observable clinical factors (pain, function, and imaging scores) is data accessibility: there is often no invasive testing needed and readouts are readily available in the clinic. When considering timing of DMOAD intervention and identification of disease mechanisms, patients often do not present to clinic until they have “end-stage” disease where pain or function is severely affected. Indeed, the structural features of OA do not consistently correlate with each other or with symptoms. Patients can suffer severe pain, but show limited radiographic evidence of OA, or have late-stage radiographic OA, but limited pain. However, cross-talk between cartilage damage and pain can occur, with pain-targeting strategies (e.g., corticosteroids) leading directly or indirectly to cartilage loss [76–78].

Neither the clinical phenotype nor the combination of risk factors directly reveals mechanism of disease, preventing meaningful identification, development, and evaluation of chondroprotective strategies [79]. In fact, a number of molecular mechanisms (endotypes) may contribute to each risk factor-induced effect and clinical phenotype. While a first step in stratification for chondroprotection may utilize clinical scoring and risk factors, it is unlikely that these will be meaningful in isolation. Future studies should explore the interrelationships among structure, function, and pain and integrate molecular endotyping to generate robust stratification systems.

### Molecular Signatures

Tissue-specific mechanisms will underlie OA, some unique to an individual tissue and some common across multiple tissues and joints. Recent studies have defined OA subtypes to be bone-, cartilage-, and inflammation- or synovium-driven [80]. It is logical to expand these principles to examine the contributions of the fat pad and synovial fluid, as well as meniscus, muscle, tendon, and ligament [9]. The contribution of each joint tissue to cartilage damage will vary between patients, ultimately combining to define one of the clinical subtypes on the OA spectrum.

#### Genotypes

OA has a complex polygenic genetic architecture, with multiple alleles acting in concert to both increase and decrease risk of disease and distinguish disease subtypes. This is demonstrated through the identification of novel and non-overlapping genetic risk loci for hip and knee OA in a meta-analysis of 17,151 hip OA patients, 23,877 knee OA patients, and > 560,000 controls [81]. A further genome-wide analysis of UK Biobank data identified 15 novel signals for hip OA, 7 for knee OA, and 6 shared between hip and knee OA [82••]. A number of loci were within genes involved in cartilage development or homeostasis (*COL11A1*, *COL11A2*, *FGFR3*, *GDF5*, *TGFB1*, *IL11*). The genetic susceptibility loci showed site-specific association and shared correlation with phenotypes including obesity, BMI, bone mineral density, and age at first live birth. Furthermore, the OA-associated loci functionally affected genes in their vicinity, altering gene expression in degraded compared to intact cartilage.

#### Cells and Transcriptomics

Improved granularity in OA stratification is possible by defining the cellular and molecular basis of OA using cytometry and next-generation sequencing approaches. These add to traditional histochemistry-based approaches and offer the possibility of a new disease taxonomy. Bulk genome-wide expression analysis of chondrocytes in articular cartilage revealed two major patient subgroups, differing in complement activation, innate immune responses, and Wnt and TGFβ signaling response [83•]. Single-cell sequencing has also identified cell subsets associated with both cartilage zone and level of damage [84••]. Although many approaches are cartilage-based, plasma miRNA signatures can distinguish levels of OA cartilage damage as either significant or minimal, also indicating epigenetic mediators as disease modulators [85]. CD14+CD16+ macrophages, enriched in the synovial fluid of knee OA patients at the time of intervention, correlate with stiffness and function scores [86•]. In the synovium, RNA-Seq of synovial macrophages has also revealed two distinct subtypes of OA – an inflammatory subtype in which macrophages have a proliferative (Ki-67 positive) signature and a ‘classical’ subtype in which macrophages possess a tissue remodelling signature [87].

#### Protein, Lipid and Metabolomic Biomarkers

There has been significant focus on using biomarkers to stratify OA and correlate with clinical signs and symptoms. Investigated biomarkers include ECM components and breakdown products, cytokines, adipokines, metabolites, lipids, and enzymes in blood, serum, urine, and synovial fluid, as well as tissues of the OA joint. Differing levels of knee cartilage loss are positively correlated with serum cartilage oligomeric matrix protein and fibulin-3 levels, whereas IL-6 is associated with increased synovitis and pain [88]. The presence of IL-17 in synovial fluid may also discriminate an OA subtype with faster cartilage loss, reduced osteophytes, and increased adipokines. Increased pain in hip OA patients is associated with increased IL-6, visfatin, and leptin, while in knee OA patients, increased leptin, reduced adiponectin and reduced adiponectin–leptin ratio are associated with pain [89–91]. Given the association of OA with metabolic disease, it is feasible that lipid and metabolomic markers may define specific OA subtypes and impact disease progression as they do in cancer [92–94].

To help crystallize critical OA stratification panels, multidimensional computational strategies should help us to deconvolute and integrate the myriad of interrelationships between transcriptomic and biomarker signatures, as well as clinical signs, symptoms, and risk factors. The same approach should be applied to determining meaningful end points to assess interventions. The combinatorial information from a range of genetic, epigenetic, transcriptomic, protein, lipid, or metabolomic biomarkers is important. Different combinations may mediate different effects, and single stratifiers may not convey any meaningful effect or association. In turn, molecular or cellular endotypes may contribute to one clinical phenotype, and some clinical phenotypes may share the same endotypic contributors. An integrated approach will therefore provide a more robust framework for successful chondroprotection and clinical translation.

## Integrating Stratification with Chondroprotective Strategies

**Fig. 3:**
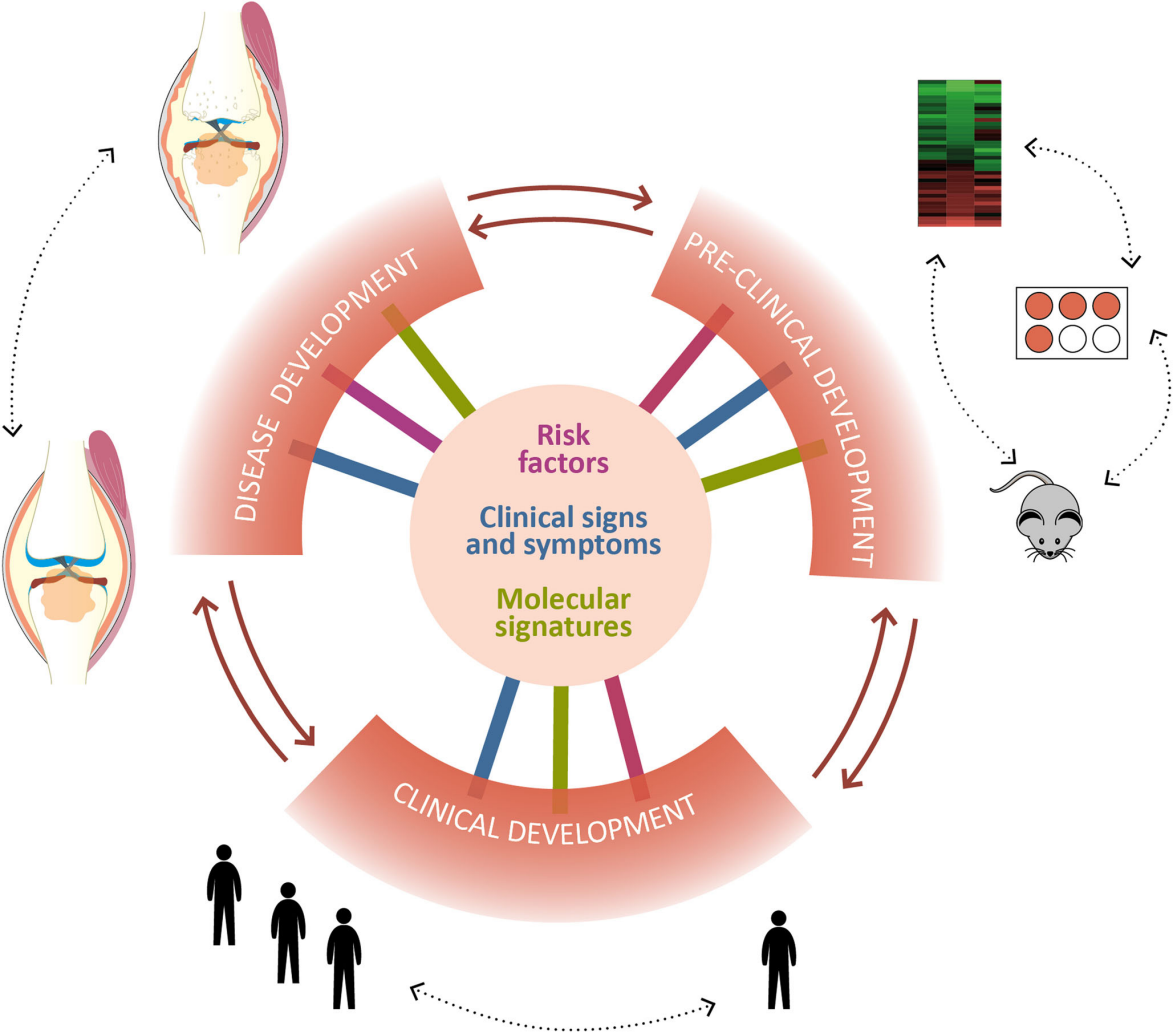
Integration of OA stratification categories with the translational cycle. The key stratification categories should be applied to studies of disease development and to pre-clinical and clinical drug development pathways. To refine critical stratification measures and identify relevant end points for pre-clinical and clinical studies, nextgeneration sequencing, “omics”, and cytometry approaches should be integrated with risk factors, clinical signs and symptoms. Tissue-based end points and stratification measures should be derived using well-phenotyped healthy and diseased tissues from the joint. Where appropriate, embedding tissue collection and analysis within enrolment and outcome stages of clinical trials would inform future studies across the translational cycle

To better deliver OA chondroprotection, phenotypes and endotypes need to be utilized intelligently—both in disease stratification and in the identification of meaningful end points. This will enable identification of pathobiological mechanisms, molecular targets, responsive groups, and markers of therapeutic efficacy. This is demonstrated in rheumatoid arthritis, where distinct transcriptomic signatures correlate with drug responses and define the clinical subtypes of disease [95]. Stratification and meaningful end points must be incorporated into all stages of the translational pipeline—in discovery, pre-clinical, and clinical studies of chondroprotection (Fig. 3).

### Pre-clinical Studies

Cellular or molecular signatures may not have functional effects on cartilage damage or OA pathobiology and may instead simply postcode a disease state or subtype. Therefore, successful delivery of chondroprotection relies on well-conducted functional studies and on the extension of cross-sectional and longitudinal studies of OA endotypes—all informed by clinical signs and risk factors. Tissues across the whole joint plus urine, blood, and synovial fluid should be incorporated. Cellular and molecular signatures of tissues obtained from diseased joints, healthy joints and joints following t herapeutic intervention are also essential. These signatures will enable identification of underlying mechanisms of disease and produce meaningful readouts to assess whether an intervention results in a “healthy” or “effectively treated” signature. The use of the spectrum of relevant tissues and fluids allows more robust endotype identification, while identifying less invasive surrogates for tissue-based signatures. Stratification panels will undergo continuous evolution, changing not only with disease subset but also with the stage of cartilage damage and other clinical readouts and risk-factors.

Studies of disease mechanisms, drug target identification, and drug testing rely on in vitro and in vivo methods, supplemented with in silico and mathematical modeling, to identify key drivers, stratification sets, and end points. Functional assays and modeling of identified biomarkers, cell types, and molecular signatures will enable validation of potential targets for chondroprotective agents and testing of therapeutic efficacy. Models and end points used in vitro and in vivo need to account for differing endotypes and changing molecular signatures associated with OA progression or cessation. As with human disease, these models should not be chondrocentric. Firstly, chondroprotection alone is not adequate for patient-beneficial changes in pain and function. Secondly, OA is a whole joint disease, with multiple studies showing significant cross-talk among bone, synovium, muscle, fat pad, meniscus, ligament, and cartilage. Finally, we should consider comorbidities, risk factors, and the mechanical environment that will all affect cellular, molecular, and functional readouts in vitro and in vivo.

For in vitro studies, well-phenotyped human tissue is the optimal source of cells and tissues. Molecular and cellular interrogation, co-cultures, and microfluidic and organ-on-a-chip platforms alone and in combination with bioreactor-driven loading will enable study of multidirectional interactions between cartilage and other critical tissues and mediators. Since the joint itself presents challenges for sampling, the development of minimally invasive biopsy methods will be necessary. The soft tissues of the joint are more amenable to biopsy, and, given their likely involvement in secreting factors that propagate OA pain and cartilage loss, research efforts may be better focused here than on interrogation of cartilage itself. However, differing cell subsets and molecular signatures in cartilage may still contribute to OA, driving diversity in disease mechanism, changes in pain and function, or response to therapeutics. Given the importance of pain and function to patients, it would be fruitful to focus on in vitro signatures that relate to these clinical readouts in order to improve the identification of potential target molecules and therapeutics.

In vivo validation is embedded within the translational pathway, and a variety of in vivo models of OA are available [96]. To establish mechanisms, targets, or drug efficacies, the multiple subtypes and risk factors of OA must be considered in the selection of an appropriate in vivo model. For example, the commonly used destabilization of medial meniscus (DMM) mouse model uses injury to induce OA in the knee of young male animals, yet only 12% of knee OA patients have post-traumatic disease and many of these patients will be female. Cartilage damage in the DMM is sex dependent, and, given the distinct pain pathways in males compared to females, stratification by sex is preferable in all in vivo models [97]. In addition, the age of mice at DMM and their diet affects disease progression and molecular response [98, 99]. This stresses that age, sex, joint site, diet, obesity, and loading regime pre- and post-disease or therapeutic intervention should be considered carefully, as should the genetic background of animals and the incorporation of comorbidity models [100]. End points in animal studies should encompass traditional cartilage readouts (e.g., OARSI scores) alongside effects on molecular signatures of other joint tissues and pain and function measures that relate to human disease.

Future work should include next-generation transcriptomic, “omic,” and cytometry platforms, in combination with multidimensional co-culture techniques, to develop high-throughput screening of chondroprotection agents. However, a clearly defined molecular signature of “healthy” or “effectively repaired” tissues will be crucial to such strategies. To this end, it is critical that future clinical trials incorporate tissue end points into their design.

### Clinical Trials

Many promising drugs have completed pre-clinical and early-phase clinical trials, but most have failed in phase III trials. It is plausible that this is due to the heterogeneity in endotypes of the patient populations within trials or to the design of targeting of patients with too advanced disease. A priori patient selection into trials requires validated inclusion criteria, including clinical markers, risk factors, and endotypes of disease to identify individuals most likely to respond to treatment [101]. Identifying molecular endotypes that predispose to OA or mark early OA is a first step in selecting patient groups within the effective therapeutic window for pharmacologic intervention—most likely before joint damage, function, and pain are too severe. Furthermore, stratification by molecular endotype for “basket” trials (one pharmacologic, multiple OA joint sites with a shared endotype) or “umbrella” trials (multiple pharmacologics targeting a molecular endotype at one joint site) may hold greater promise than traditional phase trials. Given that 31 million adults are affected by OA in the USA alone [1], successful targeting of a small subset of patients would be life changing for millions of people. For both surgical and pharmacologic interventions, reversal of all disease symptoms is unlikely. However, enabling optimal response to treatment is within reach. Clinical observations of imaging, pain, and function provide commonly used end points for clinical trials, often in combination with activity levels and patient reported outcome measures (PROMS). Combining these with meaningful molecular readouts at an accessible and relevant tissue level is also essential at trial end points where efficacy is inferred, especially given the interaction of all these factors. Without careful selection of end points, the ability to separate responders from non-responders is limited and effective (direct or indirect) chondroprotection may be missed. Furthermore, tissue readouts following clinical trials provide essential information to feed back into the discovery pipeline [102]. Finally, epidemiology studies of any clinically adopted therapeutics would enable large-scale evaluation of population-level efficacy.

## Future Look

The OA field continues to evolve, with the advent of new technologies contributing to improved strategies for therapeutic delivery and the identification of novel, unexplored targets. A reframed translational pathway, encompassing integration of stratification with discovery, pre-clinical, and clinical studies, should be applied to these advances.

### Administration, Delivery, and Release of Drugs

A particular challenge in OA is effective drug administration to ensure delivery and release at the site of action. OA is mainly localized to the particular joint affected by the disease, and many drugs, such as the MMP inhibitors, have failed due to systemic side effects. Intra-articular injections have received increased interest, as their benefits in targeted delivery compared to oral and intravenous formulations may outweigh their disadvantages. In order to decrease the frequency, and associated patient-burden, of these injections, the half-life of the drugs, as well as slow-release methods, will need to be considered.

Recent advances in delivery and release vehicles of drugs make intra-articular injections more attractive. Hydrogels, micro- and nanoparticles, and liposomes are all potential carriers for drugs, but microparticles are the most likely to provide an adequate retention time for appropriate drug release over a therapeutically useful period [103]. However, as even intra-articular approaches can affect multiple tissues, tissue-specific targeting may be necessary. A novel approach used electrostatic interactions to get drugs into the cartilage and closer to chondrocytes [104]. These cartilage-penetrating nanocarriers improve delivery and efficacy of growth factor treatment, as shown in a rat model of OA [105••]. The delivery of drugs specifically to the cartilage, where necessary, would be a major breakthrough in the field.

In addition to the use of these strategies for drugs that have already undergone development, nanomicelles can be employed to carry mRNA, as shown using the cartilage-anabolic transcription factor RUNX1, which significantly suppressed disease progression after injection into mouse OA knee joints [106]. Virus-like particles have also been coupled to recombinant nerve growth factor to create a vaccine. Pre-clinical studies showed that both prophylactic and therapeutic vaccination resulted in the attenuation of chronic pain behavior in surgically induced murine OA [107].

### Emerging Targets

Emerging targets for chondroprotection include mediators of oxidative stress, and of cholesterol and lipid metabolism, as well as nuclear receptors.

Oxidative stress occurs during disease and aging and is detrimental to many cells. Oxidative stress is evident in OA cartilage, as OA chondrocytes have increased reactive oxygen species-induced DNA damage and lipid peroxidation products. Overexpression of the antioxidant heme oxygenase-1 (HO1) was shown to protect against cartilage damage in post-traumatic and aging models of OA [108]. Oxidative stress targeting could encompass both small molecules and bioactives. S-methylisothiorea, an inhibitor of inducible nitric oxide synthase, produced anti-nociceptive and anti-arthritic effects in a rat OA model by inhibiting cartilage damage and suppressing nitric oxide in synovial fluid [109]. In addition, the bioactive sulfurophane induces HO1 and protects against DMM-induced cartilage damage [110].

Given that obesity-associated comorbidities are widely accepted risk factors for OA, it is unsurprising that cholesterol and its metabolic regulators are potential targets. High cholesterol diets alter chondrocyte cholesterol metabolism and uptake and induce more severe OA following DMM. Knockdown of CH25H–CYP7B1–RORα axis members—involved in cholesterol uptake, metabolism, and transcriptional response—decrease cartilage damage [111]. RORα, a nuclear receptor, is already a target in other diseases. Nuclear receptors are transcription factors activated by lipophilic ligands including vitamin D, glucocorticoids, retinoic acid, free fatty acids, and eicosanoids—all of which show altered expression and disease modulation in OA models and are amenable to therapeutic targeting [70]. Lipid mediators are not only pro-inflammatory like eicosanoids but also drive inflammation resolution. Resolvin D1 inhibits synovial macrophage-driven cartilage damage [112], and the resolvin precursor 17-HDHA is associated with pain but not cartilage loss [113]. These emerging targets converge on the chronic inflammatory pathways that underlie OA. Although chondroprotection is a key goal, the molecular targets should incorporate OA natural history, targeting tissues that drive cartilage loss, pain, and dysfunction in each relevant disease subtype.

## Conclusion

Significant progress has been made in understanding OA and in developing technologies to interrogate and treat this complex disease. It is now clear that chondroprotection should target all joint tissues not just cartilage. The field should continue to adopt collaborative strategies that combine cutting-edge cytometric, omics, and computational technologies. Future approaches should target the taxonomy of OA, embedding disease stratification as well as meaningful tissue, patient, and clinical end points within all stages of the translational pathway. Improved definitions of OA subtypes, meaningful end points, and targets will enable us to re-purpose or re-investigate existing therapeutics, as well as to accelerate the development of novel agents. The overarching goal for chondroprotection must be to provide the right treatment(s) for the right patient at the right time.
